# Outcomes of an HCV elimination program targeting the Viennese MSM population

**DOI:** 10.1007/s00508-021-01898-9

**Published:** 2021-06-28

**Authors:** Mathias Jachs, Teresa Binter, David Chromy, Horst Schalk, Karlheinz Pichler, David Bauer, Benedikt Simbrunner, Lukas Hartl, Caroline Schmidbauer, Florian Mayer, Robert Strassl, Mattias Mandorfer, Michael Gschwantler, Thomas Reiberger

**Affiliations:** 1 Division of Gastroenterology and Hepatology, Department of Medicine III, Medical University of Vienna, Währinger Gürtel 18–20, 1090 Vienna, Austria; 2 Vienna HIV and Liver Study Group, Medical University of Vienna, Vienna, Austria; 3 Department of Dermatology, Medical University of Vienna, Vienna, Austria; 4 Gruppenpraxis Schalk Pichler, Vienna, Austria; 5 Ihr Labor, Medical Diagnostic Laboratories, Vienna, Austria; 6 Institute of Clinical Virology, Department of Laboratory Medicine, Medical University of Vienna, Vienna, Austria; 7 Klinik Ottakring, Wiener Gesundheitsverbund, Vienna, Austria

**Keywords:** Hepatitis C, MSM, Direct acting antivirals, STD, Viral hepatitis

## Abstract

**Background and aims:**

Recent reports suggest an increasing incidence of hepatitis C virus (HCV) infections among MSM (men-who-have-sex-with-men). Early treatment with direct-acting antivirals (DAAs) achieves high cure rates and prevents further HCV transmission. We offered barrier-free HCV screening in the Viennese MSM population and immediate access to DAA treatment.

**Methods:**

In collaboration with gay health specialists, we screened for HCV seropositivity in Viennese MSM between 2019 and 2020. Barrier-free HCV-RNA-PCR tests, transient elastography (TE) and immediate access to DAA treatment were offered.

**Results:**

A total of 310 HCV-seropositive patients were identified. Of those, 145 could be contacted and 109 attended their appointment at our clinic. HIV-coinfection was highly prevalent in our cohort (*n* = 86/145; 78.9%), while pre-exposure prophylaxis (PrEP) was taken by 21.7% (*n* = 5/23) of non-HIV patients. Sexual risk behavior and (history of) intravenous drug use was reported by 32.1% and 13.8% of patients, respectively. Most MSM had already achieved sustained virological response (SVR) to previous antiviral treatment (*n* = 72, 66.1%) or experienced spontaneous clearance (*n* = 10, 9.2%). Advanced fibrosis was only detected in 3/109 (2.8%) patients. 30 MSM tested positive for HCV-RNA and DAA treatment was initiated in 29 patients – all achieved SVR.

**Conclusion:**

A targeted HCV test-and-treat program revealed a high prevalence of HCV seropositivity among Viennese MSM, potentially associated with high-risk sexual behavior and drug use. Early DAA treatment seems warranted in viremic HCV-MSM as SVR was 100%, which in turn prevents further HCV transmission.

## Introduction

Over the last years the proportion of hepatitis C virus (HCV) patients with MSM (men-who-have-sex-with men) as the main transmission route has considerably increased in Vienna [1, 2]. Recently, the proportion of MSM accounted for about 30–40% of patients presenting with untreated HCV infection at our human immunodeficiency virus (HIV)/hepatitis outpatient clinic at the Vienna General Hospital (VGH). This is in line with reports from other European centers describing a high incidence/prevalence of HCV infection [3], even in times of unrestricted access to direct-acting antiviral (DAA) treatment [4], not only in HIV-positive MSM but also in HIV-negative MSM, especially if they take pre-exposure prophylaxis (PrEP) [5, 6]. Unprotected anal intercourse and nasal and/or intravenous drug use are considered major modes of transmission for HCV in MSM, and respective risk behavior is commonly observed in the Viennese MSM population [7]. Early treatment of HCV-infected MSM has been shown to be highly effective [1], even among patients with ongoing drug use [8]. Furthermore, early treatment is cost-effective in HIV-positive MSM [9] and a cornerstone of decreasing the incidence and thus, the burden of HCV [10]. Therefore, international guidelines have recently proposed offering treatment urgently to patients with ongoing risk behavior, including MSM [11].

We have established a close collaboration with Viennese primary care physicians (PCPs) specializing in gay health care in Vienna to timely refer patients with viral hepatitis to our HIV/hepatitis center. The current policy includes a direct and short-term (usually < 7 days) referral option of patients with confirmed HCV infection (defined by detectable HCV-RNA) using a single call for an appointment at an established HCV phone line (HCV-Phone) [12] to our HIV/hepatitis clinic. Within this project, we aimed at improving HCV screening in the Viennese MSM population and offer immediate HCV treatment in order to decrease the risk for further HCV transmission.

## Methods

### Patients and study population

MSM as the designated risk group for screening were offered an HCV (antibody) test at the collaborating gay health specialists or our HIV/hepatitis center at the VGH between January 2019 and November 2020.

The test result was communicated to the patients by the PCPs or our HIV/hepatitis physicians within a few days, together with the information on the number to call or a fixed appointment for HCV-RNA-PCR test and/or treatment initiation (relying on the established HCV phone line at the VGH that has shown to facilitate referral, and thus, remove a barrier towards HCV treatment at our clinic [12]).

In addition, we actively invited all MSM patients with a history of HCV, as defined by ‘ever positive’ HCV antibody or HCV-RNA-PCR tests that were ever treated either at our collaborators or at our clinic, to a screening examination at our outpatient clinic for subsequent HCV-RNA-PCR testing.

We recorded the absolute numbers of all patients with proven HCV-seropositivity/positive HCV-RNA-PCR tests during the study period. DAA treatment was offered to all viremic patients and was based on international guidelines at the time of treatment initiation [11, 13]. In patients who underwent treatment, rates of sustained virological response 12 weeks after treatment (SVR12) were recorded.

Moreover, we screened for the presence of advanced chronic liver disease (ACLD), defined as F3/F4 fibrosis detected by vibration controlled transient elastography (TE) and routine laboratory testing.

Information on HIV status, (co)medication, e.g. opioid agonist therapy (OAT) or PrEP, and other clinically relevant parameters were gathered during the clinical interview at our outpatient clinic and complemented by a thorough search of all available medical records at our department/our collaborators.

### Laboratory tests

Routine laboratory tests were performed at the ISO-certified Department of Laboratory Medicine at the Medical University of Vienna. For HCV genotype (GT) determination and HCV-RNA quantification, VERSANT® HCV Genotype 2.0 Assay Line Probe Assay (Siemens Healthcare Diagnostics, Tarrytown, NY, USA) and the Abbott RealTime HCV assay (Abbott Molecular, Des Plaines, IL, USA) were used, respectively. The lower limit of linear quantification for HCV-RNA of the used assay was 12 IU/mL.

### Transient elastography (TE)

TE, i.e. the Fibroscan® system (Echosens, Paris, France), was used to conduct liver stiffness measurements (LSMs) as previously described [14]. The following liver stiffness cut-offs were used for staging liver fibrosis (F0-4): < 7.1 kPa for F0/F1; ≥ 7.1 kPa and < 9.5 kPa for ≥ F2; ≥ 9.5 kPa and < 12.5 kPa for ≥ F3; and ≥ 12.5 kPa for ≥ F4.25. Liver stiffness values ≥ 9.5 kPa (F3/4) denoted ACLD [14].

### Statistical analyses

Statistical analyses were performed using IBM SPSS Statistics 25 (IBM, Armonk, NY, USA) and GraphPad Prism 8 (GraphPad Software, La Jolla, CA, USA). Categorial variables were reported as the number and percentages of patients with certain characteristics. Continuous parameters were reported as mean ± standard deviation (SD) or median (interquartile range, IQR), according to the underlying distribution pattern previously determined by the Shapiro-Wilk test.

## Results

### Patient characteristics

**Fig. 1:**
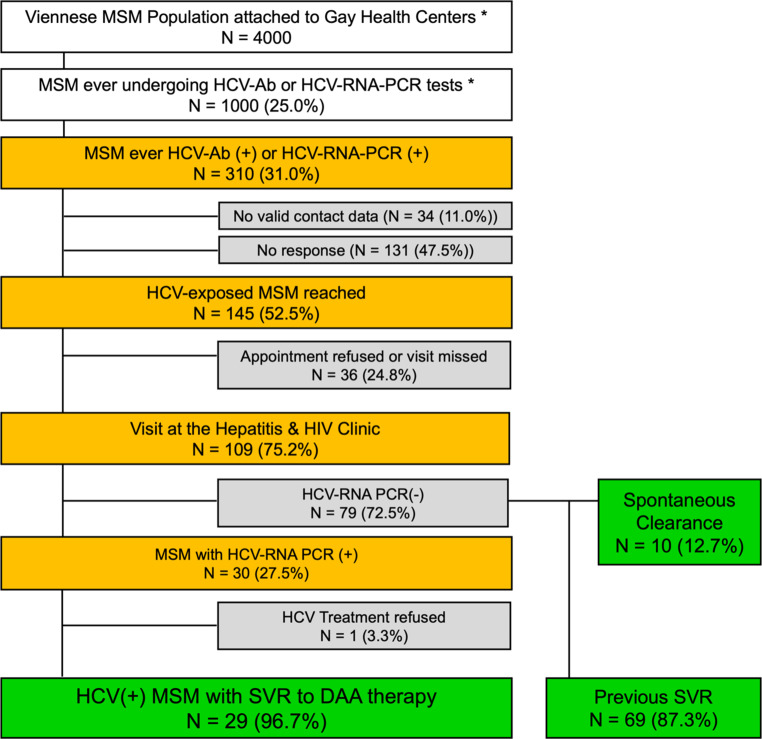
Study cohort flowchart. * Estimated numbers are based on records of our collaborating gay health specialists. *MSM* men-who-have-sex-with-men, *HCV* hepatitis C virus, *Ab* antibody, *RNA* ribonucleic acid, *PCR* polymerase chain reaction, *HIV* human immunodeficiency virus, *SVR* sustained virological response, *DAA* direct acting antiviral

Together with our collaborating gay health specialists, we identified 310 patients eligible for study inclusion due to ever positive HCV antibody or RNA-PCR tests. The total number of Viennese MSM attached to gay health specialists and the anti-HCV antibody testing rate are estimated at *n* = 4000 and at 25% (1000/4000), respectively, by gay health specialists, resulting in an estimated HCV seroprevalence rate of approximately 30% in the Viennese MSM population. While no valid contact data were available for 34/310 patients (11.0%), 145/276 (52.5%) of the remaining patients were reached by our outpatient clinic physicians or were referred to our department by our collaborators. Ultimately, 109/145 (75.2%) patients attended their fixed appointment (Fig. 1).

**Fig. 2:**
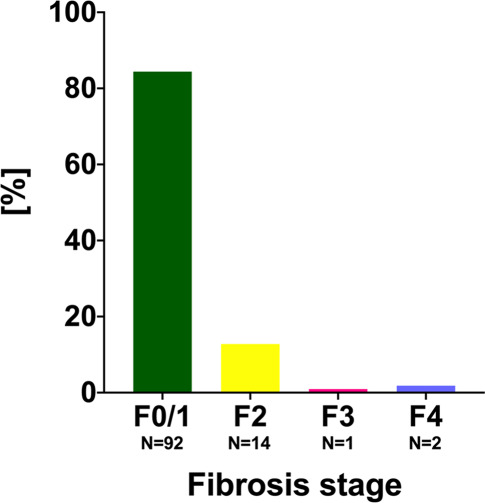
Fibrosis stages detected in the screened MSM cohort

Baseline characteristics for the 109 patients screened are summarized in Table 1. The mean age was 45.8 ± 9.9 years, and 72/109 (66.1%) had previously undergone direct-acting antiviral (DAA) or interferon-based therapy and had reached sustained virological response (SVR). HIV was present in 86/109 (78.9%) patients, and 5/23 (21.7%) HIV-negative patients took PrEP. Overall, 35/109 (32.1%) reported ongoing sexual risk behavior (defined as unprotected sexual intercourse with more than one individual of unknown disease status or under the influence of consciousness-altering drugs within the past year). The median liver stiffness was 5.1 kPa (IQR 2.4). While the vast majority of patients showed F0/1 fibrosis in vibration-controlled TE (92/109, 84.4%), 14/109 (12.8%) patients had F2 fibrosis, and evidence for advanced liver fibrosis, defined as LSM ≥ 9.5 kPa (F3/F4), was detected in 3/109 (2.8%) patients (all without decompensation), as shown in Fig. 2.

**Table 1:** Patient characteristics of all patients screened

Patient characteristic	All patients, *n* = 109
*Age, years* *±* *SD*	45.8 ± 9.9
*HIV coinfection, n (%)*	86 (78.9%)
*PrEP intake, n/non-HIV patients (%)*	5/23 (21.7%)
*Sexual risk behavior, n (%)*	35 (32.1%)
*(History of) IVDU, n (%)*	15 (13.8%)
*OAT, n (%)*	10 (9.2%)
*Previous SVR, n (%)*	72 (66.1%)
*Previous spontaneous clearance, n (%)*	10 (9.2%)
*Liver stiffness, kPa (IQR)*	5.1 (2.4)
F0/1, *n* (%)	92 (84.4%)
F2, *n* (%)	14 (12.9%)
F3, *n* (%)	1 (0.9%)
F4, *n* (%)	2 (1.8%)
*Hb, g/dL (IQR)*	15.1 (1.1)
*WBC, G/L (IQR)*	6.4 (2.9)
*PLT, G/L (IQR)*	217 (82)
*INR (IQR)*	1.0 (0.2)
*AST, IU/mL (IQR)*	25 (21)
*ALT, IU/mL (IQR)*	24 (22)
*GGT, IU/mL (IQR)*	27 (42)
*Bilirubin, mg/dL (IQR)*	0.44 (0.37)
*Albumin, mg/dL (IQR)*	46.5 (4.1)

*SD* standard deviation, *HIV* human immunodeficiency virus, *IQR* interquartile range, *IVDU* intravenous drug use, *OAT* opioid agonist therapy, *SVR* sustained virological response, *DAA* direct acting antiviral, *Hb* hemoglobin, *WBC* white blood cell count, *PLT* platelet count, *INR* international normalized ratio, *AST* aspartate aminotransferase, *ALT* alanine aminotransferase, *GGT* gamma-glutamyl transferase, *GT* genotype

### Prevalence of HCV viremia and DAA treatment initiation

Out of 109 patients, 79 (72.5%) tested negative for HCV-RNA. Importantly, 69/79 (87.3%) had been treated with DAAs or interferon-based regimens before, while 10/79 (12.7%) patients had achieved spontaneous clearance.

**Fig. 3:**
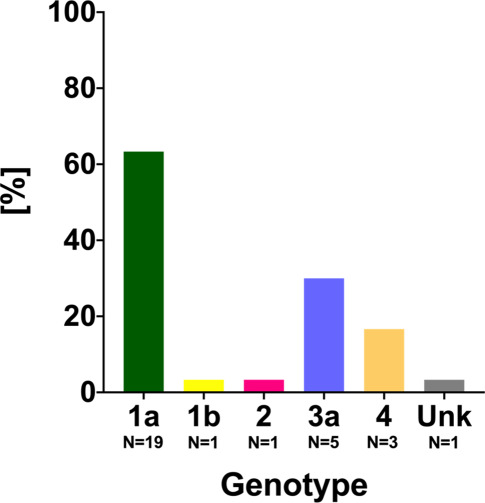
Hepatitis C virus (HCV) genotypes detected in viremic MSM. *Unk* Unknown

Moreover, 30/109 (27.5%) patients tested positive for HCV-RNA (including 3 cases of reinfections after previously achieved SVR to DAA treatment). Detailed baseline information on viremic patients is given in Table 2. Most of the viremic patients showed (recent) sexual risk behavior (73.3%). Of note, four HCV viremic patients were on daily PrEP treatment. GT1a, which was detected in 19/30 (63.4%) of patients, was the most prevalent GT, followed by GT 3a (5/30, 16.7%), and GT 4 (3/30 10.0%), as shown in Fig. 3.

**Table 2:** Characteristics of all viremic patients

Patient characteristic	Viremic patients, *n* = 30
*Age, years* *±* *SD*	41.6 ± 9.1
*HIV coinfection, n (%)*	24 (80.0%)
*PrEP intake, n/non-HIV (%)*	4/6 (66.6%)
*Sexual risk behavior, n (%)*	22 (73.3%)
*(History of) IVDU, n (%)*	5 (16.7%)
*OAT, n (%)*	1 (3.3%)
*Previous SVR, n (%)*	3 (10.0%)
*Previous spontaneous clearance, n (%)*	0 (0%)
*Liver stiffness, kPa (IQR)*	5.8 (2.7)
F0/1, *n* (%)	21 (70.0%)
F2, *n* (%)	9 (30.0%)
F3, *n* (%)	0 (0.0%)
F4, *n* (%)	0 (0.0%)
*Hb, g/dL (IQR)*	15.3 (1.9)
*WBC, G/L (IQR)*	7.2 (3.4)
*PLT, G/L (IQR)*	226 (103)
*INR (IQR)*	1.0 (0.1)
*AST, IU/mL (IQR)*	78 (94)
*ALT, IU/mL (IQR)*	175 (374)
*GGT, IU/mL (IQR)*	75 (275)
*Bilirubin, mg/dL (IQR)*	0.55 (0.36)
*Albumin, mg/dL (IQR)*	46.8 (3.4)
*HCV RNA, IU/mL (IQR)*	1.2 × 10^6^ (3.9 × 10^6^)
GT 1a, *n* (%)	19 (63.4%)
GT 1b, *n* (%)	1 (3.3%)
GT 2, *n* (%)	1 (3.3%)
GT 3a, *n* (%)	3 (10.0%)
GT 4, *n* (%)	5 (16.7%)
GT unknown, *n* (%)	1 (3.3%)
*DAA treatment initiated, n (%)*	29 (96.7%)
Glecapravir/pibrentasvir, *n* (%)	20 (69.0%)
Grazoprevir/elbasvir, *n* (%)	5 (17.2%)
Sofosbuvir/velpatasvir, *n* (%)	4 (13.8%)

*SD* standard deviation, *HIV* human immunodeficiency virus, *IQR* interquartile range, *IVDU* intravenous drug use, *OAT* opioid agonist therapy, *DAA* direct acting antiviral, *Hb* hemoglobin, *WBC* white blood cell count, *PLT* platelet count, *INR* international normalized ratio, *AST* aspartate aminotransferase, *ALT* alanine aminotransferase, *GGT* gamma-glutamyl transferase, *GT* genotype

One out of the 30 viremic patients refused treatment, thus, DAA treatment could be initiated in the remaining 29 patients. The combinations of glecapravir/pibrentasvir (G/P), grazoprevir/elbasvir (GZV/EBV) and sofosbuvir/velpatasvir (SOF/VEL) were used in 20 (69.0%), 5 (17.2%) and 4 (13.8%) patients, respectively. All patients showed excellent treatment adherence, and thus we found an SVR12 (SVR of 12 weeks or more) rate of 100%.

## Discussion

We present the results of an HCV test and treat program targeting the Viennese MSM population. Given the unrestricted access to highly effective DAA treatment [15] in Austria and the ambitious WHO goal of eliminating HCV as a major public health threat by 2030 [16], efficient screening programs for HCV infection in risk populations are urgently needed. The MSM, in which high-risk sexual behavior is highly prevalent, have emerged as a population at particular risk of acquiring HCV (re)infections, possibly being at even higher risk for infection as compared to other risk populations, e.g. people who inject drugs (PWIDs) [6], especially if they take PrEP or have already contracted HIV [5, 6]. The DAA treatment has proven to be highly efficient in MSM [1], even in patients with ongoing illicit drug use [8]. Thus, we aimed at a thorough screening program targeting the Viennese MSM population in continuous care at gay health specialists.

Extending an already established efficient collaboration with said specialists, we strived to screen for HCV in MSM and link the identified patients to our clinic and treatment using a low-threshold approach. Together with our collaborators, we were able to identify 309 patients within the Viennese MSM population who had evidence for HCV exposure by anti-HCV seropositivity or ever positive HCV-RNA viremia. Based on solid estimators, the HCV seropositivity rate in the Viennese MSM population may be as high as 30%, which is in line with reports from other European centers reporting high numbers of HCV infections in MSM [4, 5]. Relying on the collaboration with PCPs and gay health specialists and our established hepatitis hotline (HCV-phone), we invited HCV-seropositive patients to an appointment comprising TE and laboratory testing, including HCV-RNA-PCR.

We were able to screen one third of the HCV seropositive patients identified during the study period and detected HCV viremia in about 25% of screened patients. Almost all patients who were viremic agreed to DAA treatment and—due to excellent treatment adherence—achieved SVR12, which is in line with previous attempts aiming at linking high-risk populations to DAA treatment at our clinic [17]. Importantly, all patients who were viremic showed only F0/1/2 fibrosis, implicating that our test and treat approach was able to detect early stages of disease, and thus, may prevent the progression to advanced fibrosis and the development of complications. Accordingly, considerable morbidity and health care costs may have been avoided in these patients [9]. Importantly, self-reported sexual risk behavior and/or drug use was prevalent in a significant number of (viremic) patients, and thus, our program likely contributed to the prevention of further transmission by patients engaging in high-risk behavior [18].

The low proportion of patients with F3/4 fibrosis indicates a considerably lower prevalence of late presentation, as compared to a previous study that focused on screening for liver fibrosis in HIV/HCV-coinfected patients conducted from 2014–2016 at our center [19]. In this study, the prevalence of advanced liver fibrosis among viremic patients (i.e., MSM and non-MSM) attained 27%. This difference may be explained by the unrestricted access to highly effective treatment regimens, which achieve high efficacy in patients with advanced disease regardless of HIV infection [17, 20] as well as a better linkage to care and earlier presentation in the MSM population. Most of the patients screened within our program were HIV-positive and/or had already undergone DAA/interferon-based treatment, underlining the tight linkage to care within HIV-positive MSM in Vienna. Nonetheless, our results clearly indicate the need for close follow-up in HIV/HCV-coinfected patients achieving SVR, as we also detected three reinfections in those patients. In line with previous reports from other centers, HIV-negative MSM on PrEP treatment seem to be at particularly high risk of HCV infection [5, 6], which is corroborated by the fact that four out of five patients on PrEP included in our program finally tested positive for HCV-RNA; however, it is clear that this finding cannot be extrapolated to PrEP users in general.

The impact of our efforts was evidently limited by the high number of patients that could not be reached (> 50%) or refused to visit the hepatitis/HIV outpatient clinic (approximately 25% of patients reached). Therefore, the barrier to treatment might still be too high for certain sections of the screened population, although we undertook all reasonable effort to facilitate access to our institution by actively inviting all patients to a screening examination at a fixed date and at no financial cost for the patients; however, it can be assumed that the current coronavirus disease 2019 (COVID-19) pandemic also significantly impacted on our program, as access to our institution was/is still restricted and patients may hold back medical visits despite our best efforts to increase adherence in times of crisis (unpublished data).

Our findings underline the need for (i) reflex testing for HCV-RNA in HCV-seropositive patients, (ii) immediate DAA treatment initiation in viremic MSMs, and (iii) low-threshold linkage options to specialized treatment centers, e.g. via innovative solutions such as the HCV-Phone [12].

Overall, we conducted a low-threshold test and treat program for HCV in a Viennese high-risk population in urgent need of linkage to care, i.e. MSM. Relying on previously established collaborations with PCPs and gay health specialists in Vienna, as well as using our newly established HCV-Phone hotline, HCV infections within the Viennese MSM population could be efficiently detected and treated, despite limited access to care during the ongoing global pandemic. Still, a considerable proportion of HCV seropositive MSM remain to be fully evaluated and treated.
